# Side-by-side Comparison of a Picosecond 755-nm Alexandrite Laser and a Quality-switched 1064-nm Neodymium-doped Yttrium Aluminum Garnet Laser in the Treatment of Argyria

**DOI:** 10.7759/cureus.5206

**Published:** 2019-07-23

**Authors:** Emma Weiss, Kaitlyn L Streight, Christopher B Rizk, Ramsey Markus

**Affiliations:** 1 Dermatology, Yale School of Medicine, New Haven, USA; 2 Dermatology, Baylor College of Medicine, Houston, USA; 3 Dermatology, Westside Dermatology, Seattle, USA

**Keywords:** argyria, picosecond 755nm alexandrite laser, q-switched 1064nm nd:yag laser

## Abstract

Argyria is a rare but cosmetically distressing and difficult-to-treat condition for which quality-switched (Q-switched) lasers have been most commonly employed. However, at least one previous report suggests that the picosecond alexandrite laser may also serve as a successful treatment modality. Herein, we present a side-by-side comparison of a picosecond 755-nm alexandrite laser and a Q-switched 1064-nm neodymium-doped yttrium aluminum garnet (Nd:YAG) laser in the treatment of argyria. Our results reveal an equivalent success of the picosecond alexandrite when compared to the more commonly employed Q-switched Nd:YAG, suggesting that the picosecond 755-nm alexandrite laser is equally effective in the treatment of argyria.

## Introduction

Argyria is the blue-gray discoloration of the skin and mucous membranes caused by chronic exposure to silver. While asymptomatic, it is often cosmetically disfiguring and has no reliable pharmacotherapy currently available. Once considered untreatable for this reason, recent advances in laser technology have shown success in treating the condition. The quality-switched (Q-switched) lasers are the most commonly reported lasers used for the treatment of this disorder [1]. Specifically, the Q-switched neodymium-doped yttrium aluminum garnet (Nd:YAG) laser therapy can be safe and effective [1-6]. At least one previous report has suggested that the picosecond alexandrite laser is at least equivalent to other reported laser modalities in the treatment of diffuse argyria [7]. We herein report a case of a woman with diffuse argyria who was treated with both a picosecond 755-nm alexandrite laser and a Q-switched 1064nm Nd:YAG laser. The lasers were used side-by-side on her face for direct comparison.

## Case presentation

A 55-year-old Caucasian female presented to the dermatology clinic for progressive blue-grey hyperpigmentation of her face, neck, back, and extremities. She reported a five-year history of ingesting daily oral colloidal silver solution as recommended by an alternative health practitioner for her symptoms of a chronic Lyme disease. Physical exam showed diffuse, confluent blue-gray patches over the face, neck, back, extremities, and oral mucosa. Given her significant and chronic silver exposure, the clinical diagnosis of argyria was made.

**Figure 1:**
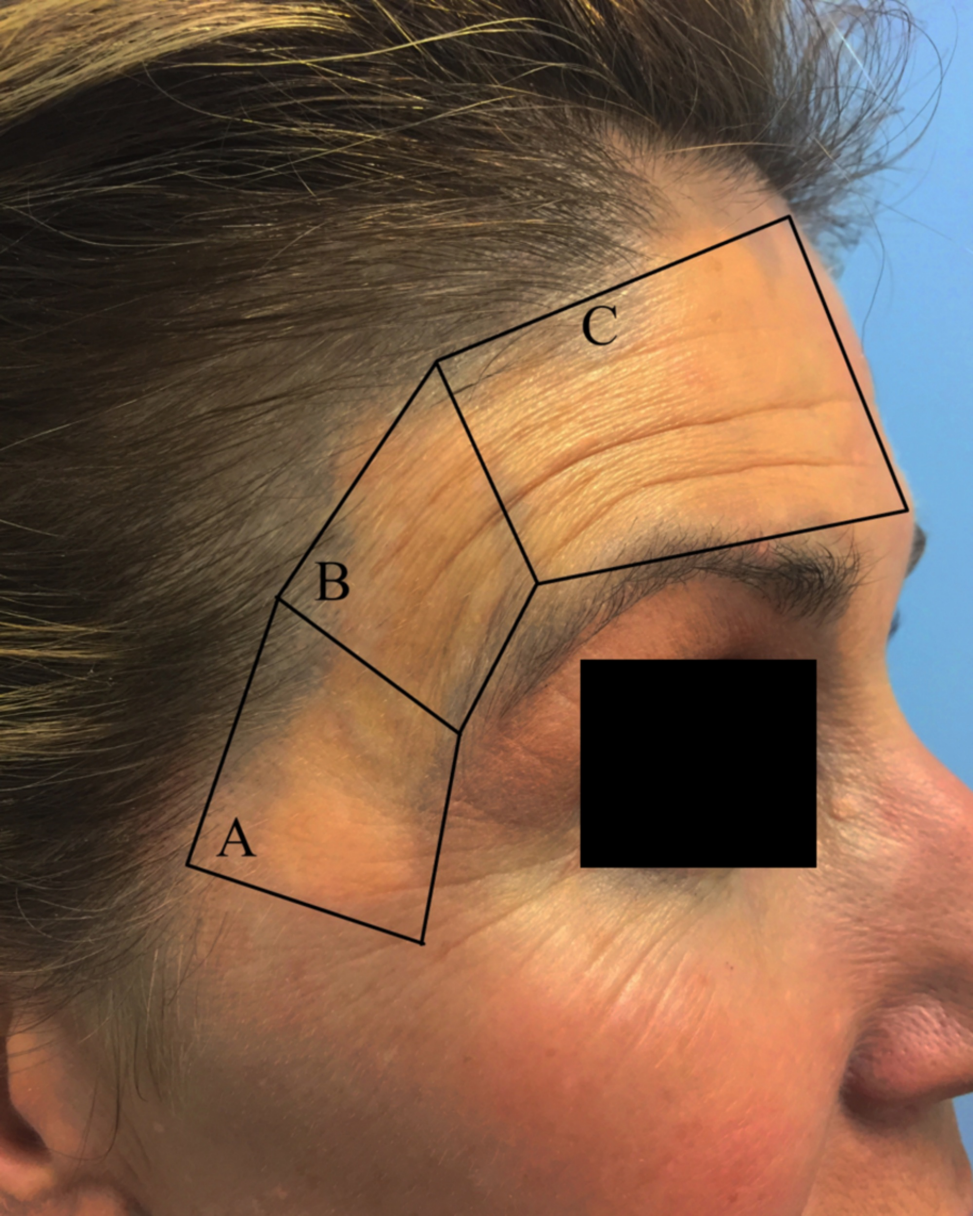
Treated regions of the right temple Area under (A) was treated with a picosecond alexandrite 755-nm laser using a fluence of 1.26 J/cm^2^ at 10 Hz, (B) with a picosecond alexandrite 755-nm laser using a fluence of 0.84 J/cm^2^ at 10 Hz, and (C) with one pass of the Q-switched 1,064-nm Nd:YAG using a fluence of 2.5 J/cm^2^ at 10 Hz.

With the patient’s permission, we proceeded with treatment using both a picosecond alexandrite 755-nm laser (PicoSure; Cynosure, Inc., Westford, MA, USA) and a Q-switched 1064nm Nd:YAG laser (QX; Fotona, Inc., Ljubljana, Slovenia) using a cold air attachment for pain control. Her right temple was treated using the picosecond alexandrite 755-nm laser. It was divided into both superior and inferior sections and treated with two different picosecond alexandrite settings. The right inferior temple was treated using a spot size of 4.5 mm, fluence of 1.26 J/cm^2^ at 10 Hz (Figure 1A). The right superior temple was treated using a spot size of 5.5 mm, fluence of 0.84 J/cm^2^ at 10 Hz (Figure 1B). Her forehead and left temple were then treated with one pass of the Q-switched 1064nm Nd:YAG using 5-mm spot size, 2.5 J/cm^2^ fluence at 10 Hz (Figure 1C).

**Figure 2:**
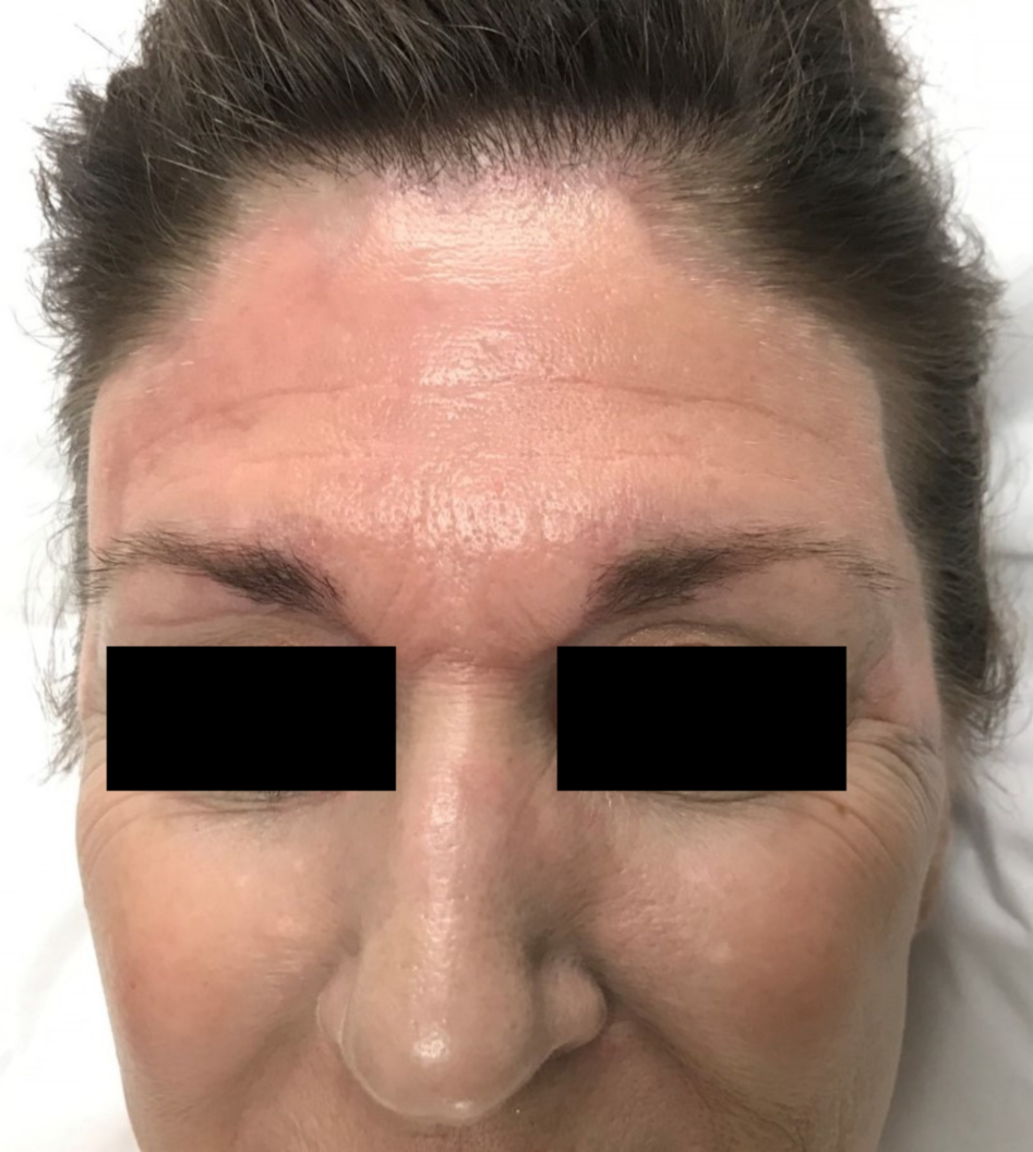
Patient immediately after procedure Improvement in the treated areas of forehead with near total clearance of the blue-grey discoloration was observed immediately following the procedure.

**Figure 3:**
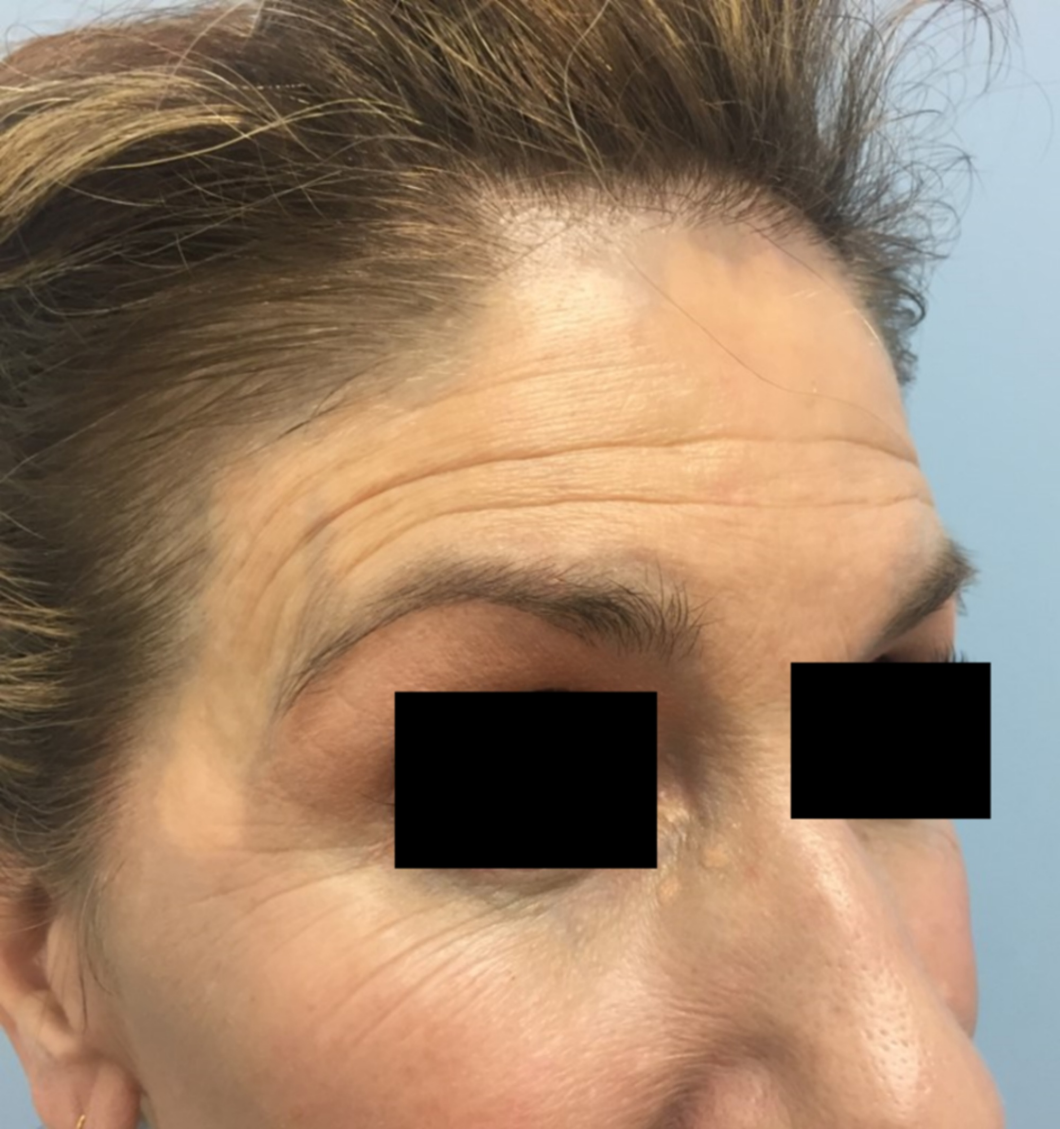
Patient at 10-week follow-up Results were maintained at ten-week follow-up, with no adverse sequelae.

The patient tolerated the procedure well without complication other than transient discomfort. Immediately, there was marked improvement in the treated areas with a near-total clearance of the blue-grey discoloration and return to normal skin pigmentation (Figure 2). Her post-operative course was unremarkable and without incident. Results were maintained at 10-week follow-up, with no adverse sequelae (Figure 3). She was exceedingly satisfied with the outcome and will continue follow-up for further treatments.

## Discussion

Argyria is a rare disorder characterized by blue-gray hyperpigmentation of the skin and mucous membranes in response to chronic silver exposure. The prevalence of argyria has diminished in recent decades due to decreased occupational exposure to aerosolized silver particles; however, the popularity of silver-containing alternative medicines has led to a resurgence of generalized argyria in recent years [1]. Argyria is distinguished pathologically by black-brown granules resulting from the deposition of silver particles into the basement membrane of eccrine glands and other dermal adnexal structures [2]. The pathophysiology is not completely understood, but the deposited silver may stimulate melanocytes to produce more melanin [8]. The discoloration is most prominent in sun-exposed areas as sunlight further stimulates the production of melanin and also catalyzes the reduction of colorless elemental silver in the dermis to silver sulfide and selenide [8]. Both silver sulfide and selenide are black-brown, leading to the characteristic color change of the condition [9]. Argyria is not associated with any additional health risks but poses significant psychosocial stress [10]. Prior to laser treatments, only limited and ineffective options were available to treat the discoloration, including dermabrasion, hydroquinone, and other depigmenting creams [3].

Several previous cases have described successful clearing of argyria’s dyschromia using a QS Nd:YAG laser [1-6]. The 1064-nm wavelength laser is poorly absorbed by melanin and water, allowing it to penetrate deeper in the dermis where the foreign silver particles reside and photoacoustically fragment the pigments into smaller pieces. Tissue macrophages then engulf the fragments and are cleared through lymphatics [3-5]. Di Giogio et al. recently described the use of the picosecond alexandrite laser in a patient with argyria who had not responded to previous treatments with several Q-switched lasers [7]. The patient had a significant correction of their skin pigment; however, follow-up was limited to one-week post-op [7]. Similar to its effectiveness for tattoo removal, the picosecond pulse width is more selective in targeting the small blue pigment [7,11]. Moore et al. postulate that Q-switched laser energy, delivered in picoseconds, may increase photomechanical and photothermal damage to pigmented structures while minimizing collateral heating of surrounding tissue as lower fluences are needed for treatment [12]. Therefore, the picosecond alexandrite may be a more efficient treatment with fewer unfavorable side effects. We add to the growing literature supporting the effectiveness of the picosecond alexandrite laser for clearing argyria’s blue-gray pigment and offer extended results at 10-week follow-up with no adverse sequelae. 

Prior to laser therapy, argyria had limited and suboptimal treatment options, often posing a significant challenge to both patients and dermatologists treating the condition. We present a case demonstrating complete resolution of argyria’s dyschromia using the picosecond alexandrite laser. The authors also show side-by-side equivalent results of the picosecond alexandrite when compared to the more commonly employed Q-switched Nd:YAG. In summary, both the Q-switched Nd:YAG and the picosecond alexandrite laser seem to be a safe and effective treatment for argyria discoloration, although additional prospective, comparative studies are warranted.

## Conclusions

Our results reveal side-by-side success in the treatment of argyria using the picosecond 755-nm alexandrite laser compared to the more commonly employed Q-switched 1064-nm Nd:YAG, suggesting that the picosecond alexandrite displays equal efficacy in the treatment of the condition. Further prospective studies comparing the safety and the efficacy of the two modalities are warranted.
